# Barriers and Facilitators to the Implementation of Family-Centered Technology in Complex Care: Feasibility Study

**DOI:** 10.2196/30902

**Published:** 2022-08-23

**Authors:** Jody L Lin, Bernd Huber, Ofra Amir, Sebastian Gehrmann, Kimberly S Ramirez, Kimberly M Ochoa, Steven M Asch, Krzysztof Z Gajos, Barbara J Grosz, Lee M Sanders

**Affiliations:** 1 Department of Pediatrics University of Utah School of Medicine Salt Lake City, UT United States; 2 John A Paulson School of Engineering and Applied Sciences Harvard University Allston, MA United States; 3 Faculty of Industrial Engineering and Management Technion – Israel Institute of Technology Haifa Israel; 4 Department of Pediatrics Stanford University School of Medicine Palo Alto, CA United States; 5 Center for Innovation to Implementation Veterans Affairs Palo Alto Health Care System Palo Alto, CA United States; 6 Primary Care and Population Health Stanford University School of Medicine Palo Alto, CA United States

**Keywords:** care coordination, implementation science, chronic illness, pediatric, family medicine, barrier, complex care, children, families, parents, care providers, chronic disease, coordination, implementation, improvement, technology, feasibility, acceptability, monitoring

## Abstract

**Background:**

Care coordination is challenging but crucial for children with medical complexity (CMC). Technology-based solutions are increasingly prevalent but little is known about how to successfully deploy them in the care of CMC.

**Objective:**

The aim of this study was to assess the feasibility and acceptability of GoalKeeper (GK), an internet-based system for eliciting and monitoring family-centered goals for CMC, and to identify barriers and facilitators to implementation.

**Methods:**

We used the Consolidated Framework for Implementation Research (CFIR) to explore the barriers and facilitators to the implementation of GK as part of a clinical trial of GK in ambulatory clinics at a children’s hospital (NCT03620071). The study was conducted in 3 phases: preimplementation, implementation (trial), and postimplementation. For the trial, we recruited providers at participating clinics and English-speaking parents of CMC<12 years of age with home internet access. All participants used GK during an initial clinic visit and for 3 months after. We conducted preimplementation focus groups and postimplementation semistructured exit interviews using the CFIR interview guide. Participant exit surveys assessed GK feasibility and acceptability on a 5-point Likert scale. For each interview, 3 independent coders used content analysis and serial coding reviews based on the CFIR qualitative analytic plan and assigned quantitative ratings to each CFIR construct (–2 strong barrier to +2 strong facilitator).

**Results:**

Preimplementation focus groups included 2 parents (1 male participant and 1 female participant) and 3 providers (1 in complex care, 1 in clinical informatics, and 1 in neurology). From focus groups, we developed 3 implementation strategies: education (parents: 5-minute demo; providers: 30-minute tutorial and 5-minute video on use in a clinic visit; both: instructional manual), tech support (in-person, virtual), and automated email reminders for parents. For implementation (April 1, 2019, to December 21, 2020), we enrolled 11 providers (7 female participants, 5 in complex care) and 35 parents (mean age 38.3, SD 7.8 years; n=28, 80% female; n=17, 49% Caucasian; n=16, 46% Hispanic; and n=30, 86% at least some college). One parent-provider pair did not use GK in the clinic visit, and few used GK after the visit. In 18 parent and 9 provider exit interviews, the key facilitators were shared goal setting, GK’s internet accessibility and email reminders (parents), and GK’s ability to set long-term goals and use at the end of visits (providers). A key barrier was GK’s lack of integration into the electronic health record or patient portal. Most parents (13/19) and providers (6/9) would recommend GK to their peers.

**Conclusions:**

Family-centered technologies like GK are feasible and acceptable for the care of CMC, but sustained use depends on integration into electronic health records.

**Trial Registration:**

ClinicalTrials.gov NCT03620071; https://clinicaltrials.gov/ct2/show/NCT03620071

## Introduction

Defined by high service needs, high resource use, and functional disability, children with medical complexity (CMC) represent a disproportionately high share of pediatric care use but receive poor quality care when compared to their noncomplex counterparts [1-6]. CMC often require care coordination across multiple health care systems with a large care team that includes professional care providers, adult caregivers, and community agencies. Care coordination through multidisciplinary care teams centered around a patient-centered and family-centered medical home may improve outcomes for CMC but can be resource-intensive [7,8]. Moreover, many CMC access care across multiple health systems, receive care in resource-limited settings, and do not live adjacent to a tertiary pediatric center where many of these clinics are based, making scalability of these innovative teams difficult. Health care that is centered around shared goal-setting is a commonly proposed approach to coordinate care for CMC to improve clinical decision-making, family engagement, and health outcomes [9,10]. Although prior studies have deployed multidisciplinary teams to create shared care plans, few studies exist for effective and scalable tools to facilitate shared goal setting [8,11]. For children with noncomplex chronic conditions (eg, asthma, type 1 diabetes), mobile health technologies may provide efficacious ways to manage chronic medical conditions for children. These positive outcomes may translate to the care of CMC but to do so may also need to overcome additional challenges such as team hierarchies, loosely coupled teams, and asynchronous time scales among providers [12-14]. Many of these challenges affect the implementation of mobile health tools, which is essential for even the most efficacious tools.

In this study, we evaluated the implementation of an internet-based shared goal-setting tool (GoalKeeper) into the care of CMC. GoalKeeper is an internet-based tool developed by the study team to improve shared goal setting between parents and providers of CMC, and designed through interviews and iterative prototyping with this population. GoalKeeper consists of 2 modules: goal elicitation and tracking. The goal elicitation module is meant to be used jointly by parents and providers during a clinic visit to set family-centered goals and is shown in Figure S1 of Multimedia Appendix 1. During goal elicitation, parents and providers are prompted verbally and visually to share the screen and use verbal prompts on the screen to set goals. The first set of prompts asks for the parent’s wishes/worries/concerns for their child’s health care and the second set of prompts helps the parents and providers set specific, measurable, and timebound goals based on the wishes/worries/concerns. A third subsection of this module provides sample goals as inspiration. The tracking module includes customizable templates that providers could assign to parents to use to track their child’s symptoms and daily progress relevant to the goals they set. After setting goals with their patient’s parents, the providers could assign tracking templates relevant to these goals for parents to use in longitudinal symptom tracking during the trial. GoalKeeper was designed to be outside the electronic health record (EHR) to allow for rapid design modifications and to enable thorough assessment of effectiveness before potential future integration into the EHR. To facilitate the integration of the entered data into the EHR, the final screen of the goal-setting module also presents the data (ie, the wishes/worries/concerns and the goals) as a block text with a button to copy the text for easy pasting into the EHR. Parents and providers had distinct interfaces where they could view and input data. Providers could create new goals and tracking forms, while parents could view set goals and input data into the tracking templates. Additional details about GoalKeeper can be found in our forthcoming companion manuscripts (B Huber et al, unpublished data, 2022, and J Lin et al, unpublished data, 2021).

To illustrate how GoalKeeper works, we will consider a sample patient, Alex, a 7-year-old child with medical complexity who arrives at the clinic with his parents. In response to the first verbal prompt, his parents state they worry about Alex not attending school and not sleeping enough. At the next prompt, Alex’s parents struggle to identify a specific goal; therefore, they turn to the sample goals for inspiration. After viewing sample goals focused on child development and discussion with their provider, they set a goal that, “Alex could be more awake during school based on adjustment of seizure medicines in the next two months.” Alex’s provider assigns a tracking template to measure school attendance and quality of sleep.

Nested within a larger effectiveness trial of the tool, this study aims to assess the barriers and facilitators of implementation of the GoalKeeper tool by using the Consolidated Framework for Implementation Research (CFIR) implementation framework. The CFIR is widely used to identify barriers and facilitators of implementation, including in health communication and adult and child chronic illness [15-18]. The CFIR contains 5 domains that interact to influence implementation effectiveness: inner setting, outer setting, characteristics of the individuals involved, intervention characteristics, and implementation process, with multiple constructs nested in each domain [15].

## Methods

### Study Design

This study is a prospective study of the implementation of a novel internet-based family-centered care plan called GoalKeeper nested under a prospective, stepped-wedge trial of GoalKeeper at a tertiary children’s health system, Lucile Packard Children’s Hospital Stanford. Details about the intervention design and results from the main trial are published in upcoming companion manuscripts (B Huber et al, unpublished data, 2022, and J Lin et al, unpublished data, 2021). Information about GoalKeeper is available through data-sharing requests directed to lsanders@stanford.edu. This study was conducted in 3 phases: preimplementation, implementation, and postimplementation. We selected a 3-phase approach, as the application of implementation science throughout intervention development is associated with increased success of implementation [18]. We selected the CFIR framework owing to its flexibility in assessing both the process of implementation and the barriers and facilitators to implementation, its use in formative evaluations at the preimplementation phase, and owing to the lack of effectiveness data of the novel tool used in the trial, as proven effectiveness is a key element of other implementation frameworks, whereas our trial evaluated the effectiveness of the tool with a secondary focus on implementation [16,19].

In the preimplementation phase, we conducted user testing in 3 stages of tool development: (1) early: parent and provider focus groups; (2) mid: individual role-play sessions and interviews using screen by screen feedback, hands-on, and think-aloud; and (3) late: pilot testing at Complex Primary Care Clinic (CPCC) with parents using GoalKeeper for a month after their clinic visit, instructing them to use GoalKeeper at least 3 times a week, followed by an exit interview. Focus groups and interviews used the CFIR interview guide questions to explore barriers and facilitators to the implementation of GoalKeeper. In the implementation phase, we implemented GoalKeeper at CPCC and pediatric neurology clinics by using implementation strategies informed by preimplementation focus groups. We recruited parent participants from the clinic of each enrolled provider for 3 weeks. Each parent was asked to use GoalKeeper with their provider at their enrollment clinic visit and for 3 months after the initial visit. At the end of the study, participants completed a postimplementation semistructured interview and survey.

### Ethical Considerations

This study was approved by the Stanford University's Single Institutional Review Board (Protocol # 32161) and is registered on ClinicalTrials.gov (NCT03620071).

### Study Population

For preimplementation focus groups and interviews, we recruited parents of CMC seen at Stanford by using a convenience sample of parents at CPCC. We selected providers from clinics planned for trial recruitment, CPCC and pediatric neurology, the hospital medicine team with specialization in caring for CMC, and a provider with expertise in clinical informatics for feedback on workflow integration. For the implementation phase, we recruited medical providers (physicians, nurse practitioners, physician assistants) from 2 clinical services that see the highest proportion of CMC: CPCC and pediatric neurology (2 clinics that historically care for many CMC). All providers at these clinics were eligible for recruitment. Based on prior work, goal setting was not routine practice in these clinics [14]. From the patients seen by enrolled providers, we recruited a convenience sample of primary caregivers (eg, parents). Parents were eligible if they were aged ≥18 years, English-speaking, and with a child with medical complexity <12 years presenting for a routine (not sick) visit. Parents who did not have home access to the internet were excluded. We excluded older children, who may have the capacity to participate in decision-making, since the tool was not designed for interaction with children [20]. We defined medical complexity as meeting all of the following criteria in the past 12 months: ambulatory visits with at least 2 subspecialty providers and functional impairment due to a chronic condition [21]. Recruitment occurred solely in-person, but due to the COVID-19 pandemic, recruitment was paused between March 13 and July 20, 2020. We did not pursue remote recruitment owing to concerns of intervention fidelity because GoalKeeper was designed to be used jointly by the provider and parents during a clinical encounter.

### Measures and Outcomes

We focused on 3 of the 5 CFIR domains: intervention characteristics (GoalKeeper), inner setting (CPCC and pediatric neurology clinics), and characteristics of individuals involved (parents and providers of CMC). We used an adapted version of the CFIR interview guide focus groups and interviews to inform our approach for implementation of GoalKeeper [15,22]. The CFIR interview guide contains open-ended interview questions organized by the CFIR domain and construct. To limit interview length, the entire study team reviewed the interview guide together and selected questions based on constructs that we felt were the most relevant to our study. All focus groups and interviews were audio recorded and transcribed for subsequent review.

In the preimplementation phase, we conducted focus groups in person with study team members as observers. Focus groups included a project overview, a demonstration of the current intervention prototype, open feedback about the intervention, and semistructured questions. In the implementation phase, we collected user data from all participants, including number of goals set, types of goals set, and number of data entries after the encounter. We assessed feasibility based on the proportion of the intervention group who used GoalKeeper during the clinical encounter. In the postimplementation phase, we conducted individual exit interviews with parent and provider participants. We assessed acceptability by using a 5-point Likert scale to determine whether GoalKeeper was useful and fit into the clinic workflow.

### Analysis

For preimplementation focus groups, all study team members collectively synthesized the key facilitators, barriers, and design considerations immediately after each focus group and interview and at weekly team meetings after reviewing the transcripts. Postimplementation exit interviews were analyzed using the CFIR qualitative analytic approach that starts with deductive coding to apply the CFIR as a coding framework and then applies inductive methods by using open, axial, and selective coding to create new codes and ultimately themes that arose from the data [23]. Three independent coders (authors JLL, KSR, and KMO) analyzed each transcript. After coding each transcript, the coders independently rated each represented CFIR construct on a scale of –2 (strong barrier to implementation) to +2 (strong facilitator to implementation) based on the CFIR analytic approach and calculated the overall ratings for all transcripts based on the median rating. At serial coding reviews, we reviewed codes and CFIR ratings to reach consensus and generated themes based off the transcripts. We calculated means and SDs for the feasibility and acceptability measures and logged data. We compared participant characteristics between parents who were interviewed and those who were not by using chi-square tests for categorical variables and 2-sided Student *t* test for continuous variables with a significance level of *P*≤.10.

## Results

### Preimplementation Results

For the preimplementation phase, in the early design stage, we conducted 1 provider focus group and 1 parent focus group. The provider focus group consisted of 3 physicians with specialties in general pediatrics, pediatric neurology, and clinical informatics. The parent focus group consisted of 2 parents of CMC. In the middesign stage, we conducted role-play sessions with 2 providers (1 complex care, 1 hospitalist) and interviewed 3 parents and 1 neurologist. In the late design stage, 2 parents pilot-tested GoalKeeper. From these focus groups and interviews, we devised 3 implementation strategies based on those previously tested by other studies: educational materials, individual technical assistance, and automated email reminders to parent participants [22].

### Implementation Results

During the implementation phase, participants received educational materials, including paper and electronic copies of an instructional manual on GoalKeeper, with parent and provider versions. Parents also received a 5-minute in-person overview of GoalKeeper, while providers received a 30-minute overview, including a 5-minute educational video on using GoalKeeper during a clinic visit. A member of the study team was available for in-person individual technical assistance in clinic and reachable by email or phone outside of clinic but did not attend the clinic visit with intervention participants. Parent participants received automated weekly email reminders to log into GoalKeeper and track their progress on the goals that were set. Email frequency was set at 1 week by default, but the reminder frequency could be modified by parents, including turning reminders off.

We enrolled 11 providers and 35 parents (15 from complex care and 20 from neurology) in the intervention arm of the trial. The providers were mostly physicians (9/11, 82%), mostly female (7/11, 64%), and pediatric neurologists (6/11, 54%). Parents had a mean age of 38.3 (SD 7.8) years, were mostly female (28/35, 80%), primarily identified as White (17/35, 49%), almost half identified as of Hispanic ethnicity (16/35, 46%), with a mean household size of 4.1 (SD 1.0), were married (23/35, 66%), and had at least some college education (86%). Children of participants had a mean age of 5.9 (SD 3.8) years, 18 (51%) identified as White, 16 (46%) as Hispanic, 25 (71%) were followed by a neurologist, 20 (57%) had technology dependence, and 20 (57%) had neurodevelopmental delay. A total of 16 parents were lost to follow-up: 7 at the 1-month follow-up and 9 at the 3-month follow-up; 9 providers and 19 parents completed exit surveys, and 9 providers and 18 parents (7 CPCC, 11 neurology) completed exit interviews. Parent, provider, and child characteristics can be found in Tables 1-3, respectively.

During the initial clinic visit at the start of the study, 34 parent-provider dyads completed goal setting with GoalKeeper. For the 1 parent-provider dyad who did not complete goal setting, the provider decided in the visit that the parent was not a good fit to participate in the trial as the patient was revealed during the clinic visit to have an acute medical issue that needed to be the focus of the entire visit.

**Table 1:** Parent participant characteristics.

Characteristics		Total (N=36)	Interviewed (n=19)	Not interviewed (n=17)
Age (years), mean (SD)		38.7 (7.8)	40.4 (8.4)	36.9 (7.0)
Sex (female), n (%)		28 (78)	15 (79)	13 (77)
**Race/ethnicity, n (%)**				
	Caucasian	17 (47)	11 (58)	6 (35)
	African American	2 (6)	0 (0)	2 (12)
	American Indian	1 (3)	1 (5)	0 (0)
	Asian	3 (8)	2 (11)	1 (6)
	Hispanic	14 (39)	4 (21)	10 (59)
	Other	1 (2.8)	1 (5)	0 (0)
**Insurance, n (%)**				
	Medicaid	13 (36)	4 (21)	9 (53)
	Private	18 (50)	11 (58)	7 (41)
	State Children’s Health Insurance Program	1 (3)	0 (0)	1 (6)
	Medicare	5 (14)	3 (16)	2 (12)
	Other	7 (19)	6 (32)	1 (6)
	Don’t know	1 (3)	0 (0)	1 (6)
	Decline to answer	1 (3)	0 (0)	1 (6)
Household size, mean (SD)		4.12 (1.0)	4.0 (1.1)	4.3 (0.9)
**Marital status, n (%)**				
	Single	9 (25)	3 (16)	6 (35)
	Married	23 (63)	13 (68)	10 (59)
	Living with partner	1 (3)	1 (5)	0 (0)
	Single, divorced	1 (3)	1 (5)	0 (0)
**Education level, n (%)**				
	>9th grade	1 (3)	0 (0)	1 (6)
	Some high school	1 (3)	1 (5)	0 (0)
	High school diploma	4 (11)	1 (5)	3 (18)
	Some college	10 (28)	6 (32)	4 (24)
	College degree	10 (28)	6 (32)	4 (24)
	Advanced degree	7 (19)	4 (21)	3 (18)
**Home internet access (select all), n (%)**				
	Laptop	24 (67)	12 (63)	12 (71)
	Tablet/e-reader	15 (42)	7 (37)	8 (47)
	Smartphone	26 (72)	14 (74)	12 (71)
	Mobile phone	7 (19)	1 (5)	6 (35)

**Table 2:** Provider characteristics (n=11).

Characteristics		Value, n (%)
Sex (female)		7 (64)
**Specialty**		
	General pediatrics	5 (46)
	Pediatric neurology	6 (54)
**Degree**		
	Doctor of medicine	9 (82)
	Doctor of osteopathic medicine	1 (9)
	Nurse practitioner	1 (9)

**Table 3:** Child characteristics.

Characteristics			Total (N=36)		Interviewed (n=19)		Not interviewed (n=17)
Age (years), mean (SD)			5.8 (3.7)		5.3 (3.9)		6.3 (3.7)
Sex (female), n (%)			16 (44)		8 (42)		8 (47)
**Race/ethnicity, n (%)**							
	Caucasian	18 (50)		12 (63)		6 (35)	
	African American	1 (3)		0 (0)		1 (6)	
	Asian	5 (14)		3 (16)		2 (12)	
	American Indian	1 (3)		1 (5)		0 (0)	
	Hispanic	16 (44)		7 (37)		9 (53)	
	Other	2 (6)		1 (5)		1 (6)	
**Children with special health care needs screener, n (%)**							
	Needs or uses prescription medicines	30 (83)		14 (74)		16 (94)	
	Needs or uses more medical care than usual	31 (86)		16 (84)		15 (88)	
	Functional limitations more than usual	29 (81)		15 (79)		14 (82)	
	Needs or uses special therapies	31 (86)		17 (90)		14 (82)	
	Needs or uses treatment for emotional/developmental/behavioral issues	20 (56)		12 (63)		8 (47)	
**Subspecialists, n (%)**							
	Cardiology	12 (33)		6 (32)		6 (35)	
	Neurology	27 (75)		14 (74)		13 (77)	
	Pulmonology	18 (50)		10 (53)		8 (47)	
	Development	20 (56)		12 (63)		8 (47)	
	Gastroenterology	22 (61)		10 (53)		12 (71)	
	Occupational therapy	25 (69)		14 (74)		11 (65)	
	Speech therapy	15 (42)		9 (48)		6 (35)	
	Physical therapy	25 (69)		14 (74)		11 (65)	
	Other	13 (36)		8 (42)		5 (29)	
**Technology dependence, n (%)**							
	Ventriculoperitoneal shunt	4 (11)		2 (11)		2 (12)	
	Gastrostomy tube	14 (39)		7 (37)		7 (41)	
	Tracheostomy	2 (6)		0 (0)		2 (12)	
	Other: vagal nerve stimulator^a^	3 (8)		3 (16)		0 (0)	
	None	15 (42)		7 (37)		8 (47)	
**Neurodevelopmental delay, n (%)**							
	Intellectual disability	18 (50)		9 (47)		9 (53)	
	Cerebral palsy	8 (22)		4 (21)		4 (24)	
	Visual impairment	7 (19)		4 (21)		3 (18)	
	Hearing deficit	2 (6)		0 (0)		2 (12)	
	None	15 (42)		7 (37)		8 (47)	

^a^Participants who were interviewed were more likely to have a child with a vagal nerve stimulator (*P*=.08).

### Postimplementation Results: Feasibility and Acceptability

Of the 19 parents who completed the exit survey, 13 (68%) parents responded that they would recommend GoalKeeper to other parents. Only 1 respondent would not, while others were undecided; 6 (67%) providers would recommend GoalKeeper to other providers. Providers commented that they would have wanted GoalKeeper integrated into the EHR. Parents would have wanted more options for reminders for use of the tool, 12 (86%) parents felt the tool was easy to use, 6 (75%) providers found GoalKeeper useful, and 4 (50%) providers felt GoalKeeper fit into their workflow. More details on the survey results are summarized in Table S2 of Multimedia Appendix 1.

From data use logs of participant use of GoalKeeper, parents and providers set a median of 2.5 (range 0-4) goals per initial clinic visit, and providers assigned a median of 3 (range 0-5) tracking templates to each parent participant. Each provider saw a median of 2.5 (range 0-10) parent participants. After the initial clinic visit, parents input information into GoalKeeper a median of 0 (range 0-19) times and providers viewed tracked data a median of 0.5 (range 0-2) times throughout the study period. The patterns of tool use based on the number of tracking templates created are summarized in Figure S3 of Multimedia Appendix 1. We conducted a post hoc analysis of correlation between the number of tracking templates used and the number of times parents entered data into the tracking templates and found a Pearson correlation coefficient of 0.41 (*P*=.01); 19 parents (8 CPCC and 11 neurology) did not enter any data into the tracking templates, while 13 parents (4 CPCC and 9 neurology) entered data 1-10 times, and 3 parents all from CPCC entered data over 10 times. Of the parents who were interviewed, 6 (1 CPCC and 5 neurology) did not enter any data, while 8 (2 CPCC and 6 neurology) entered data 1-10 times, and 3 (all CPCC) entered data over 10 times.

### Postimplementation Results: Barriers and Facilitators

Participant exit interviews covered CFIR domains of intervention characteristics, inner setting, and characteristics of individuals involved and their related constructs. From these interviews, we categorized each barrier and facilitator under a CFIR domain and construct based on topic and rated each CFIR construct that was represented in the interviews. Participant knowledge and beliefs about goal setting were facilitators to implementation with a rating of +1 whereas adaptability and compatibility were barriers, with each receiving a rating of –1. The complete ratings are given in Table 4. Limited quotes are provided in the text with additional quotes found in Table 5. There were no statistically significant differences in parent or child characteristics between those who were interviewed and those who were not except for participants who were interviewed were more likely to have a child with a vagal nerve stimulator (*P*=.08).

**Table 4:** Consolidated Framework for Implementation Research ratings by construct and participant type.

Domain mean ratings (scale, –2: strong barrier; +2: strong facilitator)		Parents (n=19)	Providers (n=9)
**Inner setting**			
	Tension for change: the degree to which stakeholders perceive the current situation as intolerable or needing change	1	0
	Compatibility: the degree of tangible fit between meaning and values attached to the intervention by involved individuals, how those align with individuals’ own norms, values, and perceived risks and needs, and how the intervention fits with existing workflows and systems	–1	–1
**Characteristics of individuals**			
	Knowledge and beliefs: individual’s attitudes toward and value placed on intervention as well as familiarity with facts, truths, and principles related to the intervention	1	1
	Individual state of change: characterization of the phase an individual is in, as he or she progresses toward skilled, enthusiastic, and sustained use of the intervention	1	0
**Intervention characteristics**			
	Relative advantage: stakeholder’s perception of the advantage of implementing the intervention versus an alternative solution	0	0
	Complexity: perceived difficulty of implementation, reflected by duration, scope, radicalness, disruptiveness, centrality, and intricacy and number of steps required to implement	1	0
	Adaptability: the degree to which an intervention can be adapted, tailored, refined, or reinvented to meet local needs	–1	–1

**Table 5:** Sample quotes and main themes for implementation.

			Parents		Providers	
**Facilitators**						
	**Parents and providers**					
		Goal-centered care is important to the care of children with medical complexity (tension for change)		“...I thought, ‘This is interesting. I think this is going to be good.’ Setting up the goals and giving me something on my side to kind of think about and work toward.” (Parent 1227, age 57 years, some college)		“...I think it’s important because sometimes the physician’s goals are not the same as the family’s goals. And so shared decision making doesn’t always happen.” (Provider 39, neurology)
		Reminders are helpful, and more flexibility around reminder frequency would have improved use; providers wanted limited reminders (individual state of change)		“...Mostly when I get emails from you and stuff and it also reminds me too that I need to check her goals and everything I have on there.” (Parent 1494, age 29 years, high school diploma)		“...And kind of the structured way of kind of connecting, whether it’s weekly, or daily, or monthly, or whatever it is. To keep on something that is important for the family. I think it is important. So that might be useful.” (Provider 33, complex primary care clinic)
		The tool helped facilitate goal-centered care (knowledge and beliefs)		“...There are so many things I need to take care of. But then, when it comes to zeroing down to the main thing that matters, in that way, Goalkeeper was very helpful for me.” (Parent 1510, age unknown, college degree)		“...And so in theory, this is a really great and I totally think that this is the way as a physician you should be thinking about it and trying to see what your family’s goals are so that you can understand what it is that they want you to help with.” (Provider 69, neurology)
	**Parents only**					
		The tool could be accessed from anywhere, but 2 parents still used other tools (relative advantage)		“...I always used to bring it in my own binder to the doctor and it’s just a lot to carry rather than when I just have my cell phone all the time.” (Parent 1476, age 38 years, some college)		N/A^a^
	**Providers only**					
		The tool should be used at end of visit to fit into clinic workflow (compatibility)		N/A		“...I mean sometimes if it worked out that I had to do it in the beginning of the visit because you were in the room to help me out and things like that, I found that less desirable than if I was able to time it toward the end of the visit.” (Provider 50, complex primary care clinic)
		The tool opens doors in patient care that providers otherwise would not explore (knowledge and beliefs)		N/A		“...So what the patient or their family values the most. So what they find most important to them, often times that comes into quality of life decisions, for example. So in that discussion about eating, they might value being able to eat some or having their child being able to eat some food more than a 50% reduction in their seizure frequency, or even being seizure free. Whereas by default, normally I would-- we’re very focused on seeing if we can get to zero seizures. And knowing that that’s the relative value that the family’s putting on things is very, very important. Sometimes that would mean that maybe there is a reason why that is, or just as also just means important that there’ll be something to work on because there can be trade-offs between those two things. For example, higher doses of antiseizure medications might cause more drooling or less ability to be awake to eat. Does that make sense? So both things are valuable, but which one is more valuable to that family? And so that you can-- it helps you prioritize between those two goals or two potential actions.” (Provider 63, neurology)
**Barriers**						
	**Parents and providers**					
		The tool would have been easier to access if it was embedded in the electronic health record/MyChart (compatibility)		“...I think the disadvantages is having another thing to log into. As is evidenced by the fact that I can’t seem to get in right now I can barely keep all of my logins straight at the moment but MyChart is sticky. I have to use MyChart to keep track of-- my son has a complex medical condition, right? So between the insurance billings and keeping the appointments straight and dealing with all the different providers at Stanford, I’m tethered to MyChart.” (Parent 1150, age 45 years, advanced degree)		“...The actual use of the application is a little awkward because it requires a separate login to the website. In terms of feedback, it would be nice if that could be embedded in the chart.” (Provider 63, neurology)
		Lack of feedback and closed loop communication hindered frequent use of the tool due to feelings that the other party was not using the tool (relative advantage)		“...So right now, being able to communicate with the doctor and having them understand that there is a communication between the two of you rather than on GoalKeeper it’s only a one-way email and then they’d have to call you because there was no option for them to email.” (Parent 1476, age 38 years, some college)		“...So I wouldn’t want to use it if that kind of-- for some reason we’re just not getting reliable feedback from families. If there’s not a loop there.” (Provider 50, complex primary care clinic)
	**Parents only**					
		Only providers could create and change goals, hindering parent engagement with the tool when goals became irrelevant (adaptability)		“...in the beginning when we first started, it was nice. But at the same time, as time goes by, it becomes a little bit more repetitive, especially when you’re not able to change things unless you go to the provider’s office or speak to the provider.” (Parent 1490, age 33 years, some college).		N/A
		Limited or unreliable internet access prevented constant use of the tool (adaptability)		“...I have limited internet data on my phone. The first time when I was able to access the first surveys, they were easy. The alert came. I was able to connect, and I was able to answer the questions with no problem. Again, in my case is not having access to technology when make this difficult for me.” (Parent 1668, age 53 years, some college)		N/A
	**Providers only**					
		When providers perceived they were already practicing goal-centered care, they felt the tool was redundant even if they adopted aspects of the tool into their practice (relative advantage)		N/A		“...I think it is something that I do regularly as part of our visit. So I don’t know that it’s going to change my practice or very much in terms of goal-setting.” (Provider 42, neurology)

^a^N/A: not applicable.

### Intervention Characteristics

When compared to existing tools, parents and providers felt that GoalKeeper was a better way to start goals-of-care conversations. One provider shared:

I think, personally, I always phrased it in my own clinical practice, just as, “Tell me something that is most important to you or something that is a priority for us to work on for your child right now.” Again, just trying to not actually always use the word “goal” itself. Or if you did ask what the parents’ goals were, then providing just a little bit more of an explanation. So I did think that, again, the wording of “wishes and worries” I liked. It was something new for me that was not a wording choice I had used before

Provider 57, CPCC

Parents and providers also felt that GoalKeeper facilitated teamwork during and after clinic visits. Parents also liked that GoalKeeper could be accessed from anywhere they had internet access.

All providers and most parents felt a barrier to use of the intervention was difficulty accessing the intervention because the URL link was not user-friendly, and they had to access it through a previously received email from the study team. Home internet connectivity issues also prevented some parents from using GoalKeeper in the follow-up period. Two parents perceived that the goal-setting portal being provider-driven hindered their use of the intervention because their provider was unengaged about updating the goals after the initial visit.

### Inner Setting

Almost all parents and providers felt there was a need for more shared goal setting in their current clinical care, which they felt promoted improved parent-provider engagement particularly for patients with more active complex medical issues. Providers overwhelmingly felt that parents of children who would need the provider’s long-term care to be the ideal audiences for the intervention with some providers saying that the intervention allowed them to focus on parents’ long-term goals. Parents also felt the intervention could fit parents of younger children who had developmental goals such as those with prematurity or other children with special health care needs. GoalKeeper was felt by both providers and parents to be incompatible with existing workflows because it was not integrated into the EHR and patient portal. Providers felt using the intervention during the middle or end of their visit fit better into their workflow, but that clinic time constraints make it hard to squeeze the intervention into the visit. Providers also wanted a Spanish-language form of the platform to target a population they felt is most in need of help setting goals for their child. When using the tracking module, parents overall desired provider feedback and communication about the information parents entered into the intervention with 1 parenting summarizing, “if there is no two-way communication or if there is no template or anything set up, then the value goes down” (Parent 1898, age 47 years, advanced degree). Parents admitted that at times, competing priorities for their child’s health superseded the use of GoalKeeper such as when a child became hospitalized for an issue that was not captured in the goals they set in GoalKeeper.

### Characteristics of the Individuals Involved

All participants felt that conversations centered around goal-setting were beneficial because they switched medical discussions to long-term and in the context of what is important to the family. Parents felt that the intervention shifted their mindsets by focusing them on the main concerns for their child. Parents felt that using the intervention made them more confident to articulate their concerns to their providers during the clinic visit and helped them identify to-do lists to achieve their goals for their child, with 1 parent remarking:

So I think that that’s kind of the gift of motivation because it’s like, “Oh, yes. We have some ability.” I think sometimes you look at a child who has a lot of needs and as a parent, you can get discouraged and then think, “Okay. We’ll just do whatever the doctor say.”...so I guess that’s a part of knowing that you can do some things to improve your child’s life and to reduce their sort of future medical interventions is so helpful as a lot of parents can feel helpless with these kind of situations

Parent 1368, age 37 years, college degree

Parents also remarked that GoalKeeper helped them prioritize their child’s short- and long-term goals. Parents felt supported and hopeful about the well-being of their child. However, most parents and providers did not adopt the intervention in its entirety with most participants only using the intervention during the initial clinical encounter. One provider shared that “like every habit, I think it might take many repetitions to start to want to use it regularly” (Provider 33, CPCC). Furthermore, providers were more motivated to use the intervention if they felt it opened new doors to insights about the patient that were not captured in their typical clinical practice. One provider remarked:

Well, I think there are some issues that came up and some questions I just hadn’t asked before and a lot of it focused around happiness and joy and things like that that led to conversations with families I hadn’t had before. So I think sometimes it’ll open doors for me and the preset questions might even be more so. It’d opened doors for me that I might not have opened without Goalkeeper or without asking

Provider 50, CPCC

Two providers felt GoalKeeper was redundant to their practice even though they integrated part of GoalKeeper into their future practice.

### Implementation Strategies

The 3 implementation strategies used during the study, that is, educational materials, individual technical assistance, and automated email reminders to parent participants, had varying success. The video tutorial provided to providers was found to be useful during the initial training, but few viewed the video after the training. No participant used the paper manual. Few participants used the individual technical assistant either virtually or in person, but for those who encountered technical issues during the visit, they found it useful with 1 provider commenting:

I always forgot the step where I was supposed to copy the goal and then go back to the page to add the template. So I just felt like each time you enrolled a patient, I was always turning back toward you to ask if I was clicking through it correctly to actually enter goals

Provider 57, CPCC

Parents overwhelmingly felt that the automated email reminders helped sustain their use of the intervention by reminding them of the goals they set for their child and to log into the system to use the tracking module with 1 parent remarking:

The email reminders were really great at making it easy to just log on and track it. And then it really was not time-consuming

Parent 1352, age 28, some college

## Discussion

In this pilot clinical trial of a novel internet-based goal-setting tool, we successfully implemented the tool for use during ambulatory clinic visits to facilitate goal elicitation. However, we did not succeed in the sustained use of the tool after the initial clinic visit. A key facilitator to implementation was participant value for the intervention’s stated aim: family-centered goal setting. A key barrier to implementation was inadequate integration with the EHR and patient portals. Provider use of the tool was also influenced by whether they felt the tool opened new doors to insights about the patient and their family that they were unable to get in their usual practice such as the relative priority of seizure control compared to appetite or wakefulness. Although automated email reminders were important to sustain tool use, they were insufficient. Parents and providers wanted a more dynamic way to communicate about goals through the intervention with most participants sharing that a feedback loop from the other party would have encouraged continued tool use. One aspect of the intervention design that is critically important is that the English-language and internet-based platform may exacerbate difference in care quality for minority and less-resourced populations.

Although our findings that automated email reminders and training are helpful implementation strategies are consistent with the implementation of digital health tools in adult chronic illness, we provide new insights for pediatric chronic illness care [24]. For example, we found that parents and providers both desired a feedback loop to sustain, which adds the caregiver perspective to prior studies of patient-generated health information where sustained use hinged on a tangible immediate benefit [25]. These findings also contribute practical considerations to the implementation of multidisciplinary care and family-centered care plans, particularly with regard to scalability and replicability across multiple institutions [11].

Our findings also contribute an implementation science perspective to practice transformation in digital health. We found that a lack of workflow integration hindered the compatibility of our intervention with the existing workflows of the providers. Future research should explore the use of clinical staff as mediators between providers and patients or caregivers for patient-reported information to facilitate intervention adoption and alleviate potential burdens that providers face with the introduction of a new intervention [26]. Furthermore, integration of our intervention into the workflow of clinical staff could address the needed feedback loop that our study participants desired but felt was lacking in the current form of the intervention. Having a tool that is well-integrated into clinic workflows would allow providers to revise and update goals as they evolve, the absence of which prevented parents from using the tool for a longer term in our study. A lack of workflow integration hindered other interventions across broad populations, including in advanced care planning for adults, pain management, and surgical safety [27-29]. Our intervention also was outside the electronic health portal, resulting in similar issues of workflow integration that have been observed in other digital interventions for populations with chronic conditions [30].

Although we purposefully built the intervention outside of the EHR to allow for rapid design improvements based on study results, future research should also explore integration of goal-setting tools within the EHR and patient portals to improve adoption. Recent efforts by payers to endorse the use of open application programming interfaces may help accelerate the integration of patient-generated health data into EHRs [31,32]. Such integration efforts should consider barriers found in our study, including how to represent patient-generated data and how to integrate goal-setting actions into the provider workflow [33,34]. As patients and parents gain access to provider notes, this research may guide efforts to improve patient and parent understanding, improve communication, and increase their empowerment [35,36].

Finally, language-related disparities must be addressed in any digital health intervention. Our participants emphasized the potential for an English-only internet-based intervention to exacerbate disparities in high-quality care coordination. Populations with low health literacy report lower use of digital health tools, which may widen the gaps in care quality they already experience owing to low health literacy [37,38]. Although 90% of the adults in the United States use the internet, fewer than 2 in 3 adults who identify as Hispanic or African American have broadband access at home with similar patterns based on lower income and education level [39]. Moreover, preference for languages other than English is associated with a decreased use of digital health tools for patient-provider communication [40]. Therefore, future work in implementation science of digital health tools should aim to understand the modifiable factors that influence the adoption by patients and families with preferences for languages other than English.

Our study should be interpreted in the context of a few limitations. The preimplementation phase used feedback from a small sample of parents and providers, which may have biased the selection of implementation strategies. Given the small number of providers practicing at each clinic, we limited feedback to a few providers to preserve an adequate provider sample for the trial. This study was conducted in a single academic medical center, which may not be reflective of the practice at other institutions caring for CMC. Not all parent participants participated in the exit interview, which may have introduced selection bias, but aside from the presence of the vagal nerve stimulator, there were no statistically significant differences in these 2 populations. Overall, our study period was quite short. Thus, the long-term use of our intervention, particularly on a repeat clinic visit, was not observed. The success of long-term implementation should be assessed with future studies.

### Conclusion

Family-centered technologies like our intervention can be successfully implemented into ambulatory primary and subspecialty care. However, long-term adoption rests on integration into the EHR and patient portal as well as adaptation of tools for users who prefer languages other than English.

## Appendix

Multimedia Appendix 1 Supplemental files.

## Abbreviations

CFIR: Consolidated Framework for Implementation Research
CMC: children with medical complexity
CPCC: Complex Primary Care Clinic
EHR: electronic health record
