# Supplementary material for: Barriers and Facilitators to the Implementation of Family-Centered Technology in Complex Care: Feasibility Study
Source: J Med Internet Res. 2022 Aug 23;24(8):e30902. doi: 10.2196/30902 (PMC9449827; doi:10.2196/30902)
Supplement: Multimedia Appendix 1 [file jmir_v24i8e30902_app1.docx]

**Figure S1. GoalKeeper goal elicitation module**

**
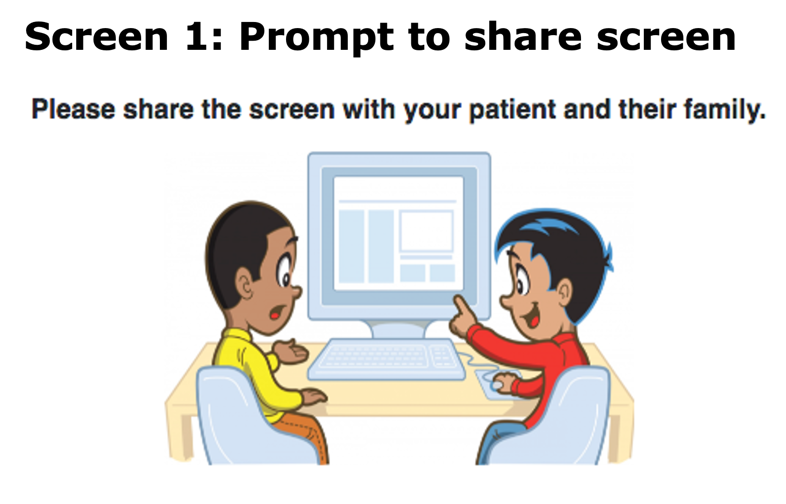

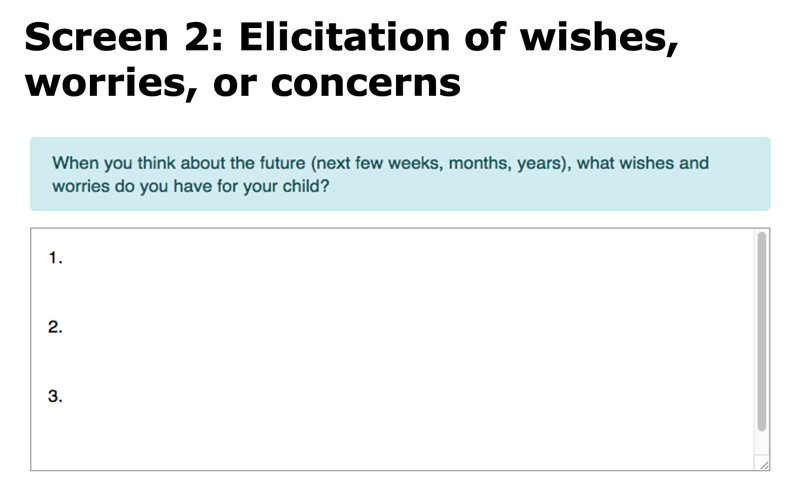

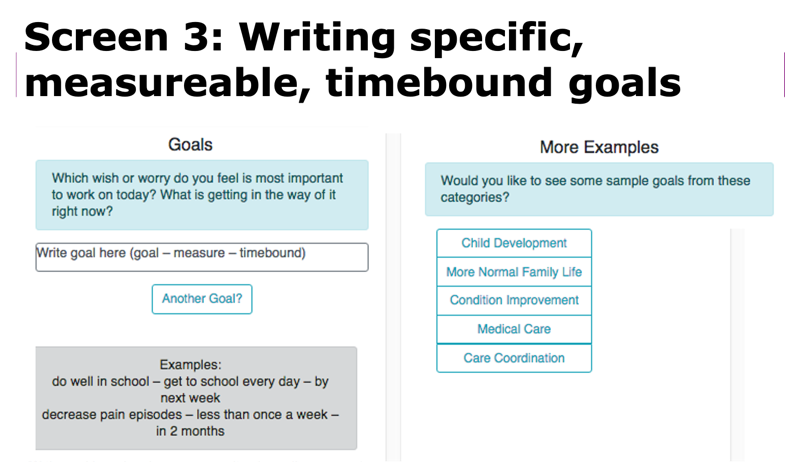
**

**Table S2. User survey results for parent and provider participants**

| **Parent survey questions** | **Agree/**  **Strongly Agree** | **Disagree/**  **Strongly disagree** | **Undecided** |  |
| --- | --- | --- | --- | --- |
| Overall, the GoalKeeper tool is useful. | 9 | 2 | 8 |  |
| I would use the GoalKeeper tool in the future. | 11 | 3 | 5 |  |
| I would recommend GoalKeeper to others | 13 | 1 | 5 |  |
| GoalKeeper helped me think about the kinds of goals I would like to set for my child. | 15 | 0 | 4 |  |
| GoalKeeper helped me and my child's doctor talk about goals for my child. | 10 | 4 | 5 |  |
| It was easy to set up an account. | 9 | 1 | 9 |  |
| It was easy to enter my goals. | 14 | 2 | 2 |  |
| It was easy to track the goals I set with my child's doctor. | 12 | 2 | 4 |  |
| **Provider survey questions** | | | | |
| Overall, the GoalKeeper tool is useful. | 7 | 1 | 1 |  |
| Using GoalKeeper helps me start important conversations with my patients' families | 6 | 1 | 1 |  |
| Using GoalKeeper helps me start important conversations about the future with my patients' families | 7 | 1 | 1 |  |
| Using GoalKeeper fits within my typical workflow | 3 | 6 |  |  |
| Using GoalKeeper helps me and my patients' families to set actionable goals | 6 | 1 | 2 |  |
| I found the example goals in GoalKeeper to be helpful. | 8 | 0 | 1 |  |
| It was easy to enter my patient's goals in GoalKeeper. | 8 | 1 | 1 |  |
| It was easy to track my patient's goals in GoalKeeper. | 7 | 1 | 1 |  |
| Would you recommend GoalKeeper to others? (yes/no) | 6 |  |  |  |

**Figure S3. Patterns of tool use based on number of tracking templates created**
